# Serum copper, zinc and metallothionein serve as potential biomarkers for hepatocellular carcinoma

**DOI:** 10.1371/journal.pone.0237370

**Published:** 2020-08-28

**Authors:** Yasuyuki Tamai, Motoh Iwasa, Akiko Eguchi, Ryuta Shigefuku, Kazushi Sugimoto, Hiroshi Hasegawa, Yoshiyuki Takei

**Affiliations:** Department of Gastroenterology and Hepatology, Mie University Graduate School of Medicine, Mie, Japan; Texas A&M University, UNITED STATES

## Abstract

**Background:**

Copper (Cu) and zinc (Zn) are essential nutrients and cofactors of enzymatic reactions with their binding partner. Metallothionein (MT) plays an important role in protecting against heavy metals and oxidative injury, however it may also portend drug resistance and a worse prognosis for hepatocellular carcinoma (HCC) patients. The aim of this study was to determine the amount of Cu, Zn, Cu/Zn and MT in evaluating a group of patients with HCC, including those treated with lenvatinib.

**Methods:**

We enrolled 175 patients with HCC (139 men, 36 women; mean age 71.1 years; hepatitis C virus n = 85, hepatitis B virus n = 19, hepatitis C virus and hepatitis B virus n = 2, non-alcoholic steatohepatitis n = 39, alcohol n = 25, others n = 5; Child-Pugh A n = 141, Child-Pugh B n = 30, Child-Pugh C n = 4; Barcelona clinic liver cancer (BCLC) stage 0 n = 38, stage A n = 56, stage B n = 39, stage C n = 38, stage D n = 4). We evaluated the associations between Cu, Zn and MT. The study outcome was liver cancer-specific survival. Moreover, we treated 12 HCC patients with lenvatinib and investigated the changes in MT during lenvatinib therapy.

**Results:**

The serum level of Cu was positively correlated with alanine aminotransferase and the BCLC stage. The serum level of Zn decreased concordant with liver disease progression. Patients with a Cu/Zn ratio≥0.999 had significantly improved rates of survival when compared to patients with a Cu/Zn ratio<0.999 (45.3 vs. 30.1 months, p<0.001). MT was significantly correlated with the Cu/Zn ratio and increased after the administration of lenvatinib. Using multivariate Cox regression analyses, it was determined that the Cu/Zn ratio (hazard ratio [HR]: 1.442, p = 0.008), alpha-fetoprotein (HR: 1.000, p<0.001) and BCLC stage (HR: 2.087, p<0.001) were independent predictors of survival.

**Conclusions:**

The Cu/Zn ratio could serve as a useful predictive marker for survival in cases of HCC. MT levels increased in HCC patients receiving lenvatinib therapy, and maybe a predictor of reduced survival.

## Introduction

Hepatocellular carcinoma (HCC) is the third leading cause of cancer-related death worldwide [1]. Various systems have been proposed for predicting the survival rate of patients with HCC [2, 3]. It is widely known that the prognosis of patients with HCC depends on the overall tumor burden and hepatic functional reserve.

Copper (Cu) and zinc (Zn) are essential nutritional metals required for the function of numerous enzymatic molecules active in human cell metabolic pathways. Cu is required for cell proliferation and tumor angiogenesis [3]. An impairment of Zn and/or Cu function has been observed in different stages of liver disease [4]. Excess Cu in the liver was the significant factor associated with the presence of HCC in a cohort of patients with hepatitis C [5]. In contrast, Zn deficiency was reported to be associated with increased liver fibrosis [6, 7] and hepatocarcinogenesis in patients with hepatitis C-related liver cirrhosis [8]. Recently, Hiraoka et al. reported that Zn deficiency is an independent prognostic factor for early stage HCC patients infected with viral hepatitis who are receiving antiviral therapy [9]. The Cu/Zn ratio has been observed to be elevated in patients with hepatitis, liver cirrhosis or HCC and appears to be correlated with the overall severity of liver disease [4, 10, 11]. In addition, it is recognized that the Cu/Zn imbalance leads to increased cellular oxidative stress [12]. Recently, Fang et al. reported that a higher Cu/Zn ratio was associated with a reduced HCC rate of survival in their prospective cohort study [13]. However, majority (88%) of the HCC samples used in their study have hepatitis B virus (HBV) infection and the association between Cu/Zn and survival was prominent only among hepatitis B negative patients.

Metallothionein (MT) is a low molecular weight, cystein-rich protein that is highly induced in response to different environmental stressors, including metal ions such as Cu and Zn, as well as cytokines and free radicals [13]. MT plays a critical role in heavy metal detoxification and acts as an antioxidant [14, 15]. In contrast, it is reported that MT expression is a prognostic factor for tumor progression and drug resistance (sorafenib) in a variety of malignancies, HCC being an exception [16–18].

However, no reports to date have presented a detailed description of the association between serum Cu/Zn ratio and MT level with regard to the survival rate of liver disease patients with HCC, as well as serum MT levels in response to lenvatinib treatment in HCC patients. In this study, we investigate the utility of the Cu/Zn ratio and/or MT level as a predictor of survival in HCC patients. Serum obtained from HCC patients receiving lenvatinib were also evaluated for MT level.

## Methods

### Patients

A total of 175 patients hospitalized in the Department of Gastroenterology and Hepatology, Mie University Hospital for treatment of HCC between May 2015 and December 2016 were included. Patients who had other malignancies within the past 3 years, severe hepatic failure greater than Model for End-Stage Liver Disease score of 30, uncontrollable infection, heart failure greater than the New York Heart Association-defined category of class II, human immunodeficiency virus infection, pregnancy, or psychiatric problems were deemed to be unsuitable for clinical study. The associations between Cu, Zn, Cu/Zn ratio or MT and rate of survival were investigated retrospectively. Patients positive for hepatitis B surface antigen were diagnosed HBV infection, whereas those positive for anti-hepatitis C virus (HCV) were diagnosed HCV infection. Alcoholic liver disease defined as the presence of alcohol consumption >20–30 g/day. NASH was diagnosed based on pathological findings and/or fatty liver without any other evident causes of chronic liver diseases (viral, autoimmune, genetic, etc.). As a general rule, the follow-up examinations included routine physical examinations and biochemical tests (1–3 monthly) and diagnostic imagining studies including ultrasonography, multiphase computed tomography, or dynamic contrast-enhanced magnetic resonance imaging. Overall survival (OS) was determined as the time from the date of hospital admission to the date of final examination, or death. Performance status was evaluated using Eastern Cooperative Oncology Group (ECOG)-performance status. Hepatic functional reserve was categorized with Child-Pugh class.

Next, we measured the level of MT in the serum of patients with HCC treated with lenvatinib. A total of 12 patients hospitalized in the Department of Gastroenterology and Hepatology, Mie University Hospital for treatment of HCC between November 2018 and January 2019 were included. Lenvatinib treatment was started as a therapy in intermediate-stage HCC beyond up-to-seven criteria and Child-Pugh A or B liver function. The inclusion criteria were as follows: unresectable HCC confirmed histologically or radiologically; tumor burden beyond up-to-seven criteria; Child-Pugh class A or B liver function; and ECOG-performance status 0. For each patient, serum samples were obtained before and 4 to 24 weeks after the initiation of treatment with lenvatinib.

This study complied with the Declaration of Helsinki, and was approved by the Clinical Research Ethics Review Committee of Mie University Hospital. Written informed consent was obtained from participants prior to participation.

### HCC diagnosis and treatment

HCC was diagnosed based on an increasing course of alpha-fetoprotein (AFP), as well as typical imaging characteristics of dynamic computed tomography [19], magnetic resonance imaging [20], and/or pathological findings. HCCs were classified with Barcelona clinic liver cancer (BCLC) staging. All treatments were performed following the Japanese practical guidelines for HCC as possible [21].

### Blood samples

Blood samples were collected when patients arrived at the hospital, and albumin (Alb), total bilirubin, aspartate aminotransferase (AST), alanine aminotransferase (ALT), alkaline phosphatase (ALP), blood urea nitrogen (BUN), creatinine, total-cholesterol, white cell counts, total lymphocytes, hemoglobin, platelet count, prothrombin time, and AFP were measured. The controlling nutritional status (CONUT) score was also calculated using Alb, total lymphocyte count, and total cholesterol level [22]. Blood samples were kept at -80°C until Cu, Zn, and MT measurements were performed. Serum Cu and Zn were measured using the Metalloassay Kit (Metallogenics, Chiba, Japan), following the manufacturer’s protocol. Then, The Cu/Zn ratio was calculated. Serum MT was determined using the competitive ELISA (Frontier Institute, Hokkaido, Japan).

### Statistical analysis

Continuous variables are presented as mean ± standard deviation or median (minimum-maximum), and categorical variables are shown as numbers of patients. The continuous data were compared using Student’s *t*-test or the Mann-Whitney test. The categorical data were compared using the Chi-squared test. Relationships between variables were determined using the two-sided Pearson’s correlation coefficient. Receiver operator characteristic (ROC) curves and the corresponding area under the curve (AUC) were used to obtain cut-offs for the outcomes. The Youden index was applied to calculate the optimal cutoff point. OS was measured using the Kaplan-Meier method and compared using the log-rank test. Associations between predictor variables and OS were determined by the hazard ratio (HR) and 95% confidence interval calculated using Cox proportional hazards regression. Forward variable selection was used in the multivariate mode. A paired t-test was used for comparing values before and after lenvatinib in the same patient. All statistical analyses were performed using SPSS 21.0 software (IBM, Armonk, NY). All tests were two-tailed, and p<0.05 was considered significant.

## Results

### Patient demographics in patients with HCC

**Fig 1:**
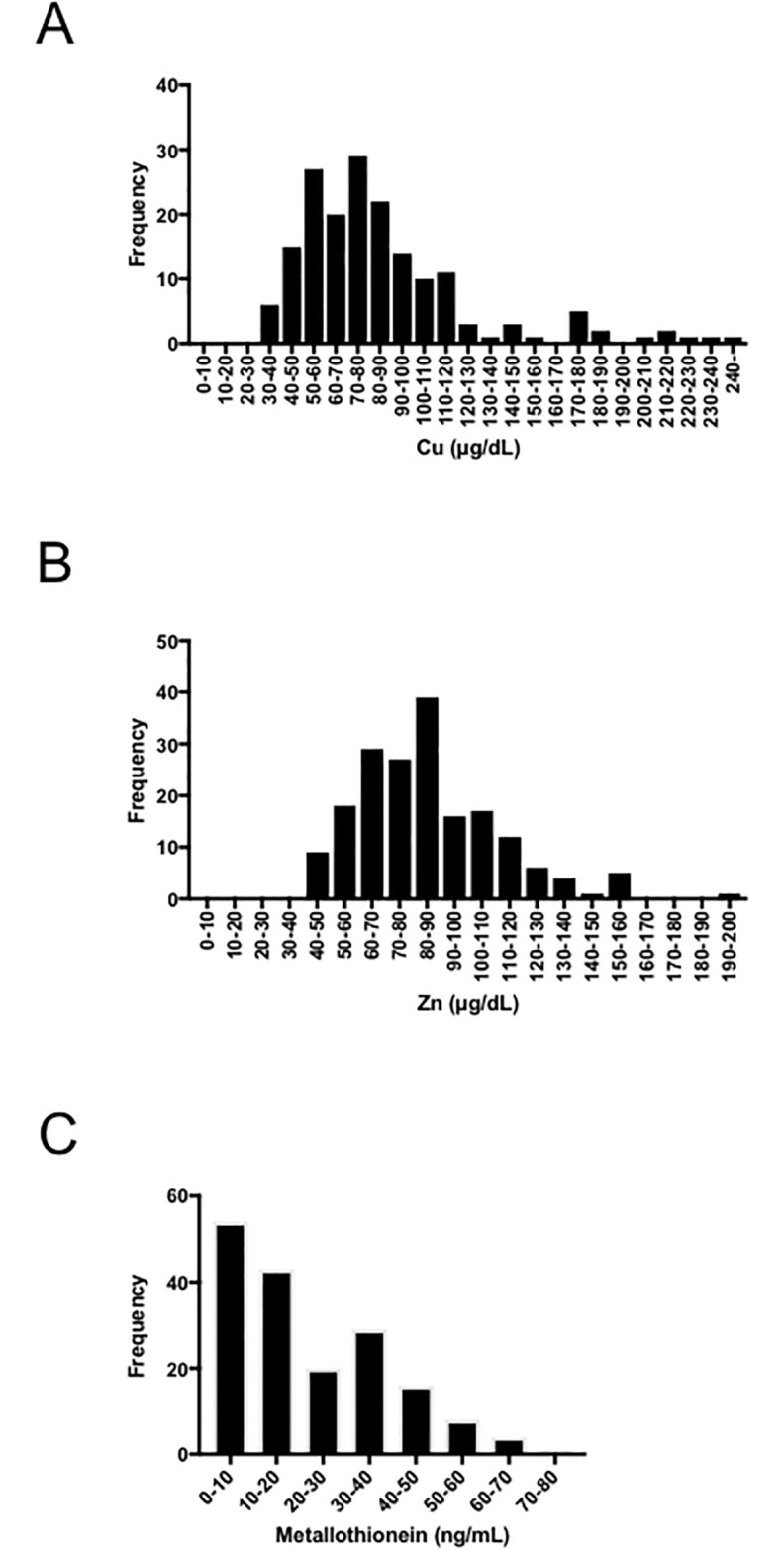
Distribution of Cu (A), Zn (B) and MT (C). Cu, copper; Zn, zinc; MT, metallothionein.

Clinical features of the 175 enrolled patients are shown in Table 1. The mean age was 71.1±8.9 years. The cohort of patients were admitted to our study based on a variety of causative agents: 19 HBV, 85 HCV, 2 HBV and HCV, 39 nonalcoholic steatohepatitis, 25 alcoholism and 5 other factors. Child-Pugh grade showed 141 patients in Class A, 30 in Class B, and 4 in Class C. BCLC staging showed 38 patients in Stage 0, 56 in Stage A, 39 in Stage B, 38 in Stage C and 4 in Stage D. The levels of Cu and Zn were normally distributed (Fig 1A and 1B) and the mean levels of Cu, Zn and Cu/Zn were 86.0±44.5 μg/dL, 85.5±26.3 μg/dL and 1.06±0.58, respectively. The frequency of Cu or Zn deficiency was 38.9% and 47.4%, respectively. The level of MT within our cohort was stepwise decreased (Fig 1C) and the mean level was 22.2±17.2 ng/mL. Nakazato, et al. reported the normal range of MT is 27–48 ng/mL (n = 200) and the MT levels in chronic hepatitis C (18 patients) were lower than healthy controls [23].

**Table 1:** Baseline clinical and biochemical profiles of patients with HCC.

	n = 175
Age, years	71.1±8.9
Gender, male/female	139/36
Body Mass Index, kg/m^2^	23.2±4.0
ECOG-performance status, 0/1/2/3/4	163/9/3/0/0
Child-Pugh grade, A/B/C	141/30/4
HCC, naive/recurrent	82/93
Etiology, HBV/HCV/HBV+HCV/NASH/alcohol/others	19/85/2/39/25/5
BCLC stage, 0/A/B/C/D	38/56/39/38/4
Albumin, g/dl	3.88±0.52
Total bilirubin, mg/dl	1.03±0.60
AST, IU/L	52.9±47.6
ALT, IU/L	38.2±27.4
ALP, U/L	383.6±228.2
Total-cholesterol, mg/dL	159.3±36.7
BUN, mg/dL	16.5±5.5
Creatinine, mg/dL	0.85±0.37
AFP, ng/ml	9 (1–1080460)
White cell counts, /μL	5237±1879
Total lymphocytes, /μL	1442.0±666.0
Hemoglobin, g/dL	12.6±2.1
Platelets, ×10^3^/μL	156.2±90.1
Prothrombin time, %	84.5±17.5
CONUT score	2.79±2.15
Cu, μg/dl	86.0±44.5
Zn, μg/dl	85.5±26.3
Metallothionein, ng/mL	22.2±17.2
Cu/Zn	1.06±0.58

^a^Data are presented as number of patients, mean ± standard deviation, or median (minimum-maximum). ECOG, Eastern Cooperative Oncology Group; BCLC, Barcelona clinic liver cancer; HCC, hepatocellular carcinoma; HBV, hepatitis B virus; HCV, hepatitis C virus; NASH, non-alcoholic steatohepatitis; AST, aspartate transaminase; ALT, alanine aminotransferase; ALP, alkaline phosphatase; BUN, blood urea nitrogen; AFP, alpha fetoprotein; CONUT, controlling nutrition status; Zn, zinc; Cu, copper

### Associations between the Cu/Zn ratio and other test values (Fig 2A–2Q)

**Fig 2:**
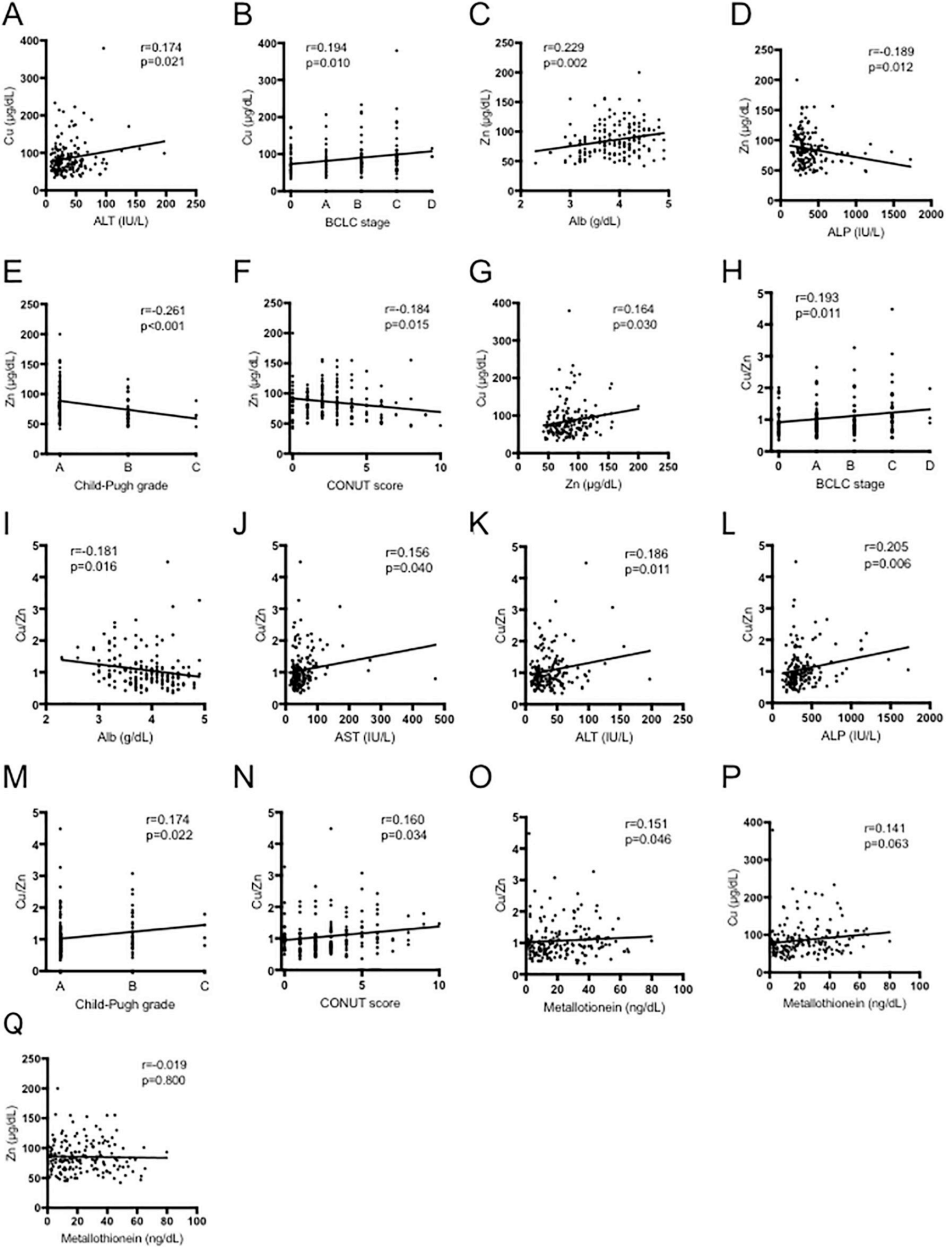
Cu and Zn are correlated with liver function and HCC progression. Correlation of (A) Cu with ALT, (B) Cu with BCLC stage, (C) Zn with Alb, (D) Zn with ALP, (E) Zn with Child-Pugh score, (F) Zn with CONUT score, (G) Zn with Cu, (H) the Cu/Zn ratio with BCLC stage, (I) the Cu/Zn ratio with Alb, (J) the Cu/Zn ratio with AST, (K) the Cu/Zn with ALT, (L) the Cu/Zn ratio with ALP, (M) the Cu/Zn ratio with Child-Pugh score, (N) the Cu/Zn ratio with CONUT score, (O) the Cu/Zn ratio with metallothionein, (P) Cu with metallothionein and (Q) Zn with metallothionein. Cu, copper; Zn, zinc; HCC, hepatocellular carcinoma; ALT, alanine aminotransferase; BCLC, Barcelona clinic liver cancer; Alb, albumin; AST, aspartate transaminase; ALP, alkaline phosphatase; CONUT, controlling nutrition status.

Serum Cu levels were positively correlated with ALT (p = 0.021; Fig 2A) and BCLC stage progression (p = 0.010; Fig 2B). Low serum Zn levels were associated with impaired liver function and nutritional status, such as hypoalbuminemia (p = 0.002), high ALP (p = 0.012), high Child-Pugh score (p<0.001) and high CONUT score (p = 0.015) (Fig 2C–2F). The levels of serum Zn were correlated with those of Cu (p = 0.030; Fig 2G). In contrast, the Cu/Zn ratio was associated with HCC progression and impaired liver function, i.e., BCLC stage (p = 0.011), Alb (p = 0.016), AST (p = 0.040), ALT (p = 0.011), ALP (p = 0.006), Child-Pugh score (p = 0.022), and CONUT score (p = 0.034) (Fig 2H–2N). Serum MT levels were correlated with the Cu/Zn ratio (p = 0.046; Fig 2O), but not Cu or Zn levels taken independently (Fig 2P and 2Q). Previous studies have shown that excess Cu and Zn deficiency were associated with HCC with viral hepatitis [5, 9]. Associations between the Cu, Zn, Cu/Zn and MT levels and other test values with or without hepatitis virus are shown in S1 or S2 Figs, respectively. The number of a significant correlation tended to increase in HCC patients with viral hepatitis when compared to HCC patients without hepatitisin this cohort.

### Patient survival

**Fig 3:**
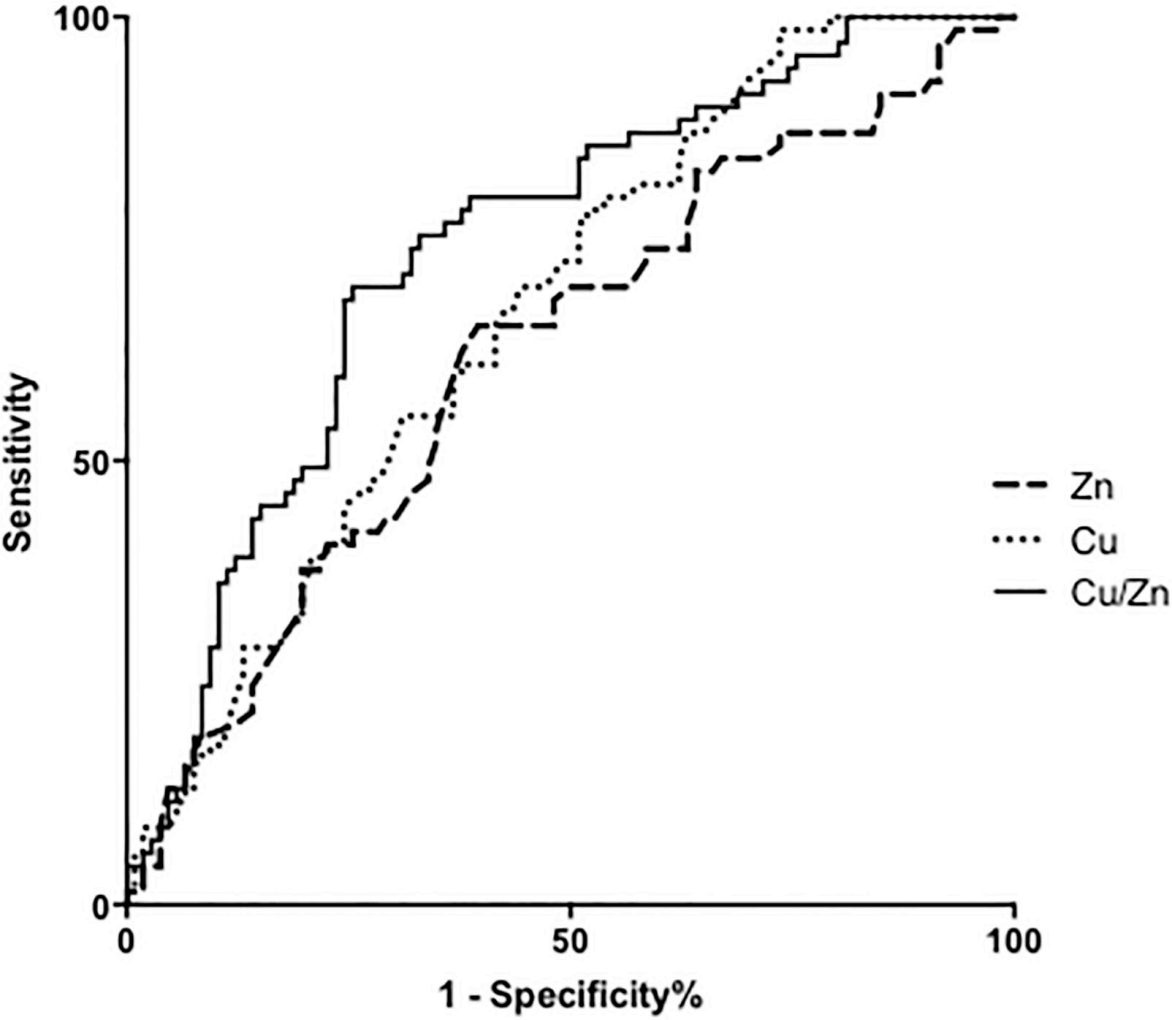
ROC curves for evaluation of overall survival of Cu, Zn, and the Cu/Zn ratio. ROC, receiver operator characteristic; Cu, copper; Zn, zinc.

69 out of 175 patients (39.4%) died in the average follow-up period of 30.2±14.1 months during our study period. The causes of death were HCC progression in 51 patients, liver failure in 7 patients, acute renal failure in 3 patients, infectious conditions, such as spontaneous bacterial peritonitis in 4 patients and gastrointestinal hemorrhage in 2 patients. The ultimate cause of death for all patients was considered liver related with the exception of two patients suffering from pancreatic cancer. ROC analyses concerning predictors of survival yielded AUC values of 0.669 (p<0.001) for Cu, 0.620 (p = 0.007) for Zn, and 0.741 (p<0.001) for the Cu/Zn ratio (Table 2). We calculated the cut off value of Cu at 68.3 μg/dL (sensitivity 0.783 and specificity 0.481), Zn at 81.1 μg/dL (sensitivity 0.604 and specificity 0.652) and the Cu/Zn ratio at 0.999 (sensitivity 0.681 and specificity 0.755) from our ROC analysis of survival curves (Fig 3).

**Table 2:** The analyses of four variables according to the ROC curve.

Variables	Sensitivity	Specificity	Optimal cutoff value	AUC	The low 95% CI	The high 95% CI	P value
Cu	0.783	0.481	68.3	0.669	0.590	0.748	<0.001
Zn	0.604	0.652	81.1	0.620	0.535	0.706	0.007
Cu/Zn	0.681	0.755	0.999	0.739	0.665	0.813	<0.001

^a^ROC; receiver operating characteristics, Cu; Copper, Zn; zinc

**Fig 4:**
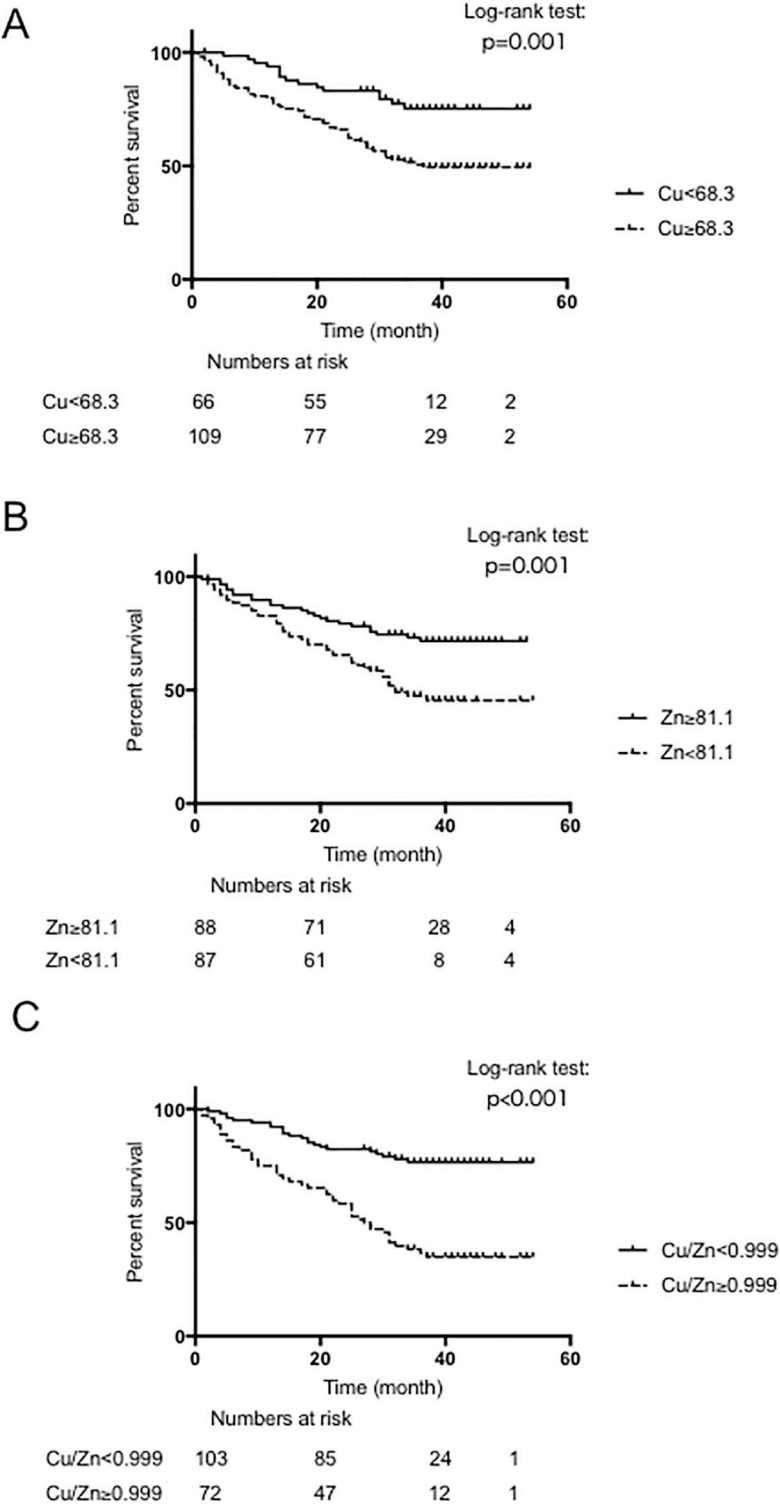
Impact of Cu (A), Zn (B) and the Cu/Zn ratio (C) on overall survival in patients with HCC. Cu, copper; Zn, zinc; HCC, hepatocellular carcinoma.

Factors of liver function, such as Alb, AST, ALP, PT and CONUT score were significantly changed in patients with a Cu/Zn ratio ≥0.999 compared to patients with a Cu/Zn ratio <0.999. Serum MT levels were also elevated in patients presenting with a high Cu/Zn ratio. Furthermore, the high Cu/Zn group included more advanced patients with untreatable HCC (Table 3). Patients with low Cu (<68.3), high Zn (≥81.1), and a low Cu/Zn ratio (<0.999) showed significantly better OS than patients with high Cu, low Zn, and a high Cu/Zn ratio, respectively (45.4 vs. 35.1 months, p = 0.001, Fig 4A; 42.6 vs. 34.4 months, p = 0.001, Fig 4B; 45.3 vs. 30.1 months, p <0.001, Fig 4C).

**Table 3:** Characteristics of patients stratified by the Cu/Zn ratio.

	Cu/Zn<0.999 n = 103	Cu/Zn≧0.999 n = 72	P value
Age, years	70.3±8.8	72.2±9.1	0.180
Gender, male/female	82/21	57/15	0.943
Body Mass Index, kg/m^2^	23.4±3.6	22.9±4.6	0.454
ECOG-performance status, 0/1/2/3/4	99/4/0/0/0	64/5/3/0/0	0.071
Child-Pugh grade, A/B/C	90/12/1	51/18/3	0.021*
HCC, naive/recurrent	46/57	36/36	0.486
Etiology, HBV/HCV/HBV+HCV/NASH/alcohol/others	14/45/2/21/17/4	5/40/0/18/8/1	0.257
BCLC stage, 0/A/B/C/D	23/44/19/16/1	15/13/20/21/3	0.004*
Albumin, g/dl	4.02±0.44	3.69±0.55	<0.001*
Total bilirubin, mg/dl	1.03±0.63	1.03±0.54	0.925
AST, IU/L	46.1±46.4	62.5±48.0	0.026*
ALT, IU/L	36.4±26.5	40.8±28.7	0.302
ALP, U/L	330.6±141.9	459.4±297.9	0.001*
Total-cholesterol, mg/dL	160.2±34.7	158.0±39.5	0.703
BUN, mg/dL	16.2±5.3	16.9±5.9	0.417
Creatinine, mg/dL	0.84±0.41	0.87±0.30	0.546
AFP, ng/mL	15950±117063	8677±39655	0.613
White cell counts, /μL	5323±1816	5113±1971	0.466
Total lymphocytes, /μL	1469.8±690.1	1402.2±632.5	0.510
Hemoglobin, g/dL	12.9±2.3	12.3±1.8	0.049*
Platelets, ×10^3^/μL	162.3±96.4	147.8±80.1	0.300
Prothrombin time, %	86.8±16.6	81.1±18.3	0.034*
CONUT score	2.45±1.87	3.28±2.43	0.016*
Zn, μg/dl	94.1±26.1	73.1±21.4	<0.001*
Cu, μg/dl	66.9±19.9	113.2±54.8	<0.001*
Metallothionein, ng/mL	18.3±14.9	27.8±18.7	0.001*
Cu/Zn	0.73±0.17	1.56±0.61	<0.001*
Treatment			0.003*
Resection, n(%)	14	6	
RFA, n(%)	53	19	
TACE, n(%)	19	28	
HAIC, n(%)	11	11	
Sorafenib, n(%)	6	6	

^a^Data are presented as number of patients, mean ± standard deviation. ECOG, Eastern Cooperative Oncology Group; BCLC, Barcelona clinic liver cancer; HCC, hepatocellular carcinoma; HBV, hepatitis B virus; HCV, hepatitis C virus; NASH, non-alcoholic steatohepatitis; AST, aspartate transaminase; ALT, alanine aminotransferase; ALP, alkaline phosphatase; BUN, blood urea nitrogen; AFP, alpha fetoprotein; CONUT, controlling nutrition status; Zn, zinc; Cu, copper; RFA; radiofrequency ablation, TACE; transcatheter arterial chemoembolization, HAIC; hepatic arterial infusion chemotherapy, BSC; best supportive care.

*p<0.05.

### Investigation of factors affecting OS

The contribution of age, gender, ECOG-Performance status, recurrent, Child-Pugh grade, creatinine, AFP, CONUT score, MT, and the Cu/Zn ratio to OS were evaluated by univariate analysis using a Cox proportional hazard model. A significant predictor of OS was the Cu/Zn ratio (p<0.001), as well as ECOG-performance status (p = 0.048), Child-Pugh grade (p = 0.021), BCLC stage (p<0.001), AFP level (p = 0.001) and CONUT score (p = 0.012). Using multivariate analysis, the Cu/Zn ratio was an independent prognostic factor (HR: 1.442, p = 0.008), while BCLC stage (HR: 2.087, p<0.001) and serum AFP level (HR: 1.000, p<0.001) were other prognostic factors (Table 4). Furthermore, we used univariate Cox regression analysis, which included Cu and Zn, and found that Cu and Zn were independent predictors of survival. However, taken alone serum Cu or Zn were not independent predictors of survival when analyzed using multivariate Cox regression analyses (S1 and S2 Tables).

**Table 4:** Univariate and multivariate analyses in patients with HCC for overall survival.

	Univariate analysis			Multivariate analysis		
	HR	95% CI	P value	HR	95% CI	P value
Age	1.016	0.989–1.044	0.248			
Gender	0.968	0.538–1.740	0.913			
ECOG-performance status	1.709	1.005–2.903	0.048*	0.789	0.622–1.869	0.789
Recurrent	1.097	0.682–1.767	0.702			
Child-Pugh grade	1.693	1.082–2.649	0.021*	0.924	0.570–1.498	0.749
BCLC stage	2.167	1.735–2.706	<0.001*	2.087	1.628–2.677	<0.001*
Creatinine	1.408	0.821–2.417	0.214			
AFP	1.000	1.000–1.000	0.001*	1.000	1.000–1.000	<0.001*
CONUT score	1.150	1.032–1.282	0.012*	1.109	0.983–1.250	0.093
Metallothionein	1.010	0.997–1.023	0.132			
Cu/Zn	1.832	1.406–2.388	<0.001*	1.442	1.099–1.892	0.008*

^a^ECOG, Eastern Cooperative Oncology Group; BCLC, Barcelona clinic liver cancer; AFP, alpha fetoprotein; CONUT, controlling nutrition status; Zn, zinc; Cu, copper.

*p<0.05.

### Patient demographics in patients with HCC treated with lenvatinib

The subjects for our study consisted of 12 patients (9 men and 3 women) with a mean age of 72.1±8.7 years (Table 5). The patients were admitted to our study based on a variety of causative agents: 2 HBV, 4 HCV, 3 alcohol consumption, 3 NAFLD. BCLC staging showed 5 patients in Stage B and 7 patients in Stage C. Child-Pugh scoring showed 11 patients in Class A and one patient in Class B. Lenvatinib was administered once a day at a dose of 8 mg for 6 patients with a body weight of less than 60 kg, and a dose of 12 mg for 5 patients with a body weight of 60 kg or more. The starting doses were reduced from 12 mg to 8 mg in one patient.

**Table 5:** Baseline clinical and biochemical profiles of patients with hepatocellular carcinoma treated with lenvatinib.

	n = 12
Age, years	72.1±8.7
Gender, male/female	9/3
Body Mass Index, kg/m^2^	22.2±3.5
Body weight, <60 kg/≥60 kg	6/6
ECOG-performance status, 0/1/2/3/4	11/1/0/0/0
Child-Pugh grade, A/B/C	11/1/0
HCC, naive/recurrent	1/11
Etiology, HBV/HCV/NASH/alcohol	2/4/3/3
BCLC stage, 0/A/B/C/D	0/0/5/7/0
Albumin, g/dl	3.91±0.47
Total bilirubin, mg/dl	0.92±0.76
AST, IU/L	49.9±43.0
ALT, IU/L	36.4±43.4
ALP, U/L	535.8±550.4
BUN, mg/dL	18.5±2.7
Creatinine, mg/dL	0.79±0.17
AFP, ng/ml	1860±4936
White cell counts, /μL	4381±1040
Hemoglobin, g/dL	11.7±1.8
Platelets, ×10^3^/μL	160.5±50.2
Prothrombin time, %	94.9±26.1
Metallothionein, ng/mL	4.67±4.56

^a^Data are presented as number of patients, mean ± standard deviation, or median (minimum-maximum). ECOG, Eastern Cooperative Oncology Group; BCLC, Barcelona clinic liver cancer; HCC, hepatocellular carcinoma; HBV, hepatitis B virus; HCV, hepatitis C virus; NASH, non-alcoholic steatohepatitis; AST, aspartate transaminase; ALT, alanine aminotransferase; ALP, alkaline phosphatase; BUN, blood urea nitrogen; AFP, alpha fetoprotein

### Changes in MT in patients with HCC treated with lenvatinib

**Fig 5:**
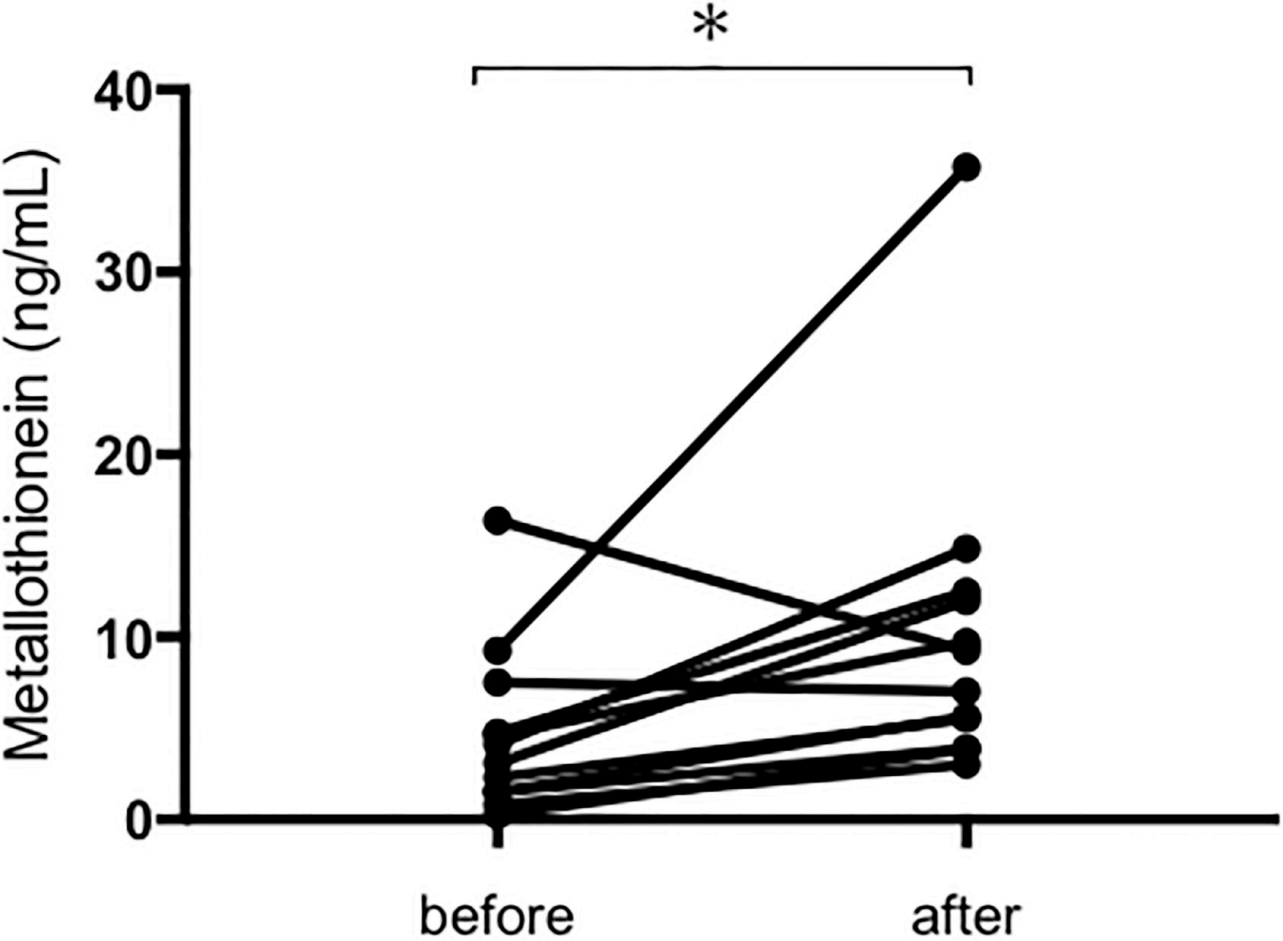
Serum MT values are presented before and after lenvatinib treatment. MT, metallothionein. * p <0.05.

We found that serum levels of MT significantly increased in patients with HCC treated with lenvatinib (median value before lenvatinib 2.7 ng/mL, median value after lenvatinib 8.1 ng/mL, p<0.05; Fig 5). At the individual level, 6 out of 12 patients presented a more than three-fold increase in MT upon receiving lenvatinib treatment.

## Discussion

Disturbances in Cu and Zn homeostasis have been frequently observed in patients with HCC. The oxidative stress potential of copper can induce an increase in free radical production and result in cellular damage [24]. In the present study, serum Cu levels were correlated with ALT. Zhang reported that serum Cu levels were significantly higher in patients with malignant hepatic tumors greater than 5 cm in size when compared to patients presenting with smaller tumors, and a significant reduction in serum Cu levels after tumor reduction was obtained through treatment [25]. We also confirmed in this study that the level of serum Cu was correlated with BCLC stage.

Serum Zn levels were associated with hepatic function tests, such as Alb, ALP and Child-Pugh grade, which is consistent with previous reports [26]. In the present study, we found that serum Zn levels were correlated with CONUT score. Zn deficiency in patients with liver disease is caused by a variety of factors, namely, inadequate oral intake, diminished hepatic extraction, portosystemic shunts and the effects of cytokines (mainly IL-6) and endotoxins [27], and we assumed these conditions lead to poor nutritional status [22, 28, 29].

Oral Zn supplementation is effective in controlling Cu balance in patients with Wilson’s disease and blocks the intestinal absorption of Cu through MT induction [30, 31]. Furthermore, MT is induced by heavy metals and oxidative injury [32]. In our cohort, serum MT was significantly correlated with the Cu/Zn ratio, but not serum Cu or Zn levels taken independently. Further study is needed related to the changes in MT expression during Zn supplementation and the association between MT and other heavy metals in patients with chronic liver disease, including HCC.

MT plays an important role in the protection against heavy metals and oxidative injury. A knockout model of MT was shown to promote hepatocarcinogenesis by superoxide production in mice, suggesting that MT is a tumor suppressor insofar as it inhibits tumorigenesis [32]. In contrast, MT is up-regulated in resistant cancer cells and is thought to be responsible for acquired chemoresistance [33]. Sun et al. reported that MT facilitates sorafenib resistance through the inhibition of ferroptosis [13]. In addition, Houessinon et al. reported that high induction of MT in the serum was indicative of poor prognosis in HCC patients treated with sorafenib [34]. In recent years, lenvatinib has become available as a single agent for the first-line treatment of patients with advanced, or unresectable, HCC [35, 36]. Predictive and prognostic markers in HCC patients treated with lenvatinib are lacking. In this study, lenvatinib was also able to induce MT in patients with HCC. Furthermore, serum MT levels were correlated with the Cu/Zn ratio and significantly elevated in patients within the high Cu/Zn ratio group. We could not certify the association between an increase in MT after lenvatinib and patient survival due to a small number of patients. The Cu/Zn ratio and/or MT may be useful in the evaluation of treatment resistance and prognosis, although future research is required.

In general, tumor stage [2], tumor markers [37, 38], Child-Pugh grade [39] and nutritional status [40] are well-known prognostic factors among HCC patients. In the present study, BCLC stage and AFP levels were independent prognostic factors in HCC patients. We further showed that the Cu/Zn ratio could be used as an independent predictor of survival. Child-Pugh grade and CONUT score did not show statistical significance as a predictor of patient survival in the present study. These results suggest that the Cu/Zn ratio might be a more accurate and only patient-related prognostic factor, although we need to increase our number of patients with Child-Pugh B and C in future studies.

Despite our important findings, there are a few limitations to the present study. First, this study was retrospective and was conducted within a single medical center. Second, the number of patients within our cohort is low. It will be important to examine a multicenter validation study in the future. Evaluation of the behavior of Cu, Zn and Cu/Zn in cirrhotic non-HCC patients is also important. In addition, the effectiveness of Zn supplementation for prolonging survival in patients with HCC should be conducted using a randomized control study.

In conclusion, the present results show that the Cu/Zn ratio appears to be a strong predictor of survival in HCC patients. Serum MT levels increase in HCC patients receiving lenvatinib, and may be associated with an overall reduction in rate of survival.

## Supporting information

S1 Fig Association between the Cu/Zn ratio and other test values with virus hepatitis. Correlation of (A) Cu with ALT, (B) Cu with BCLC stage, (C) Zn with Alb, (D) Zn with ALP, (E) Zn with Child-Pugh score, (F) Zn with CONUT score, (G) Zn with Cu, (H) the Cu/Zn ratio with BCLC stage, (I) the Cu/Zn ratio with Alb, (J) the Cu/Zn ratio with AST, (K) the Cu/Zn with ALT, (L) the Cu/Zn ratio with ALP, (M) the Cu/Zn ratio with Child-Pugh score, (N) the Cu/Zn ratio with CONUT score, (O) the Cu/Zn ratio with metallothionein, (P) Cu with metallothionein and (Q) Zn with metallothionein. Cu, copper; Zn, zinc; HCC, hepatocellular carcinoma; ALT, alanine aminotransferase; BCLC, Barcelona clinic liver cancer; Alb, albumin; AST, aspartate transaminase; ALP, alkaline phosphatase; CONUT, controlling nutrition status.
(TIFF)

S2 Fig Association between the Cu/Zn ratio and other test values without virus hepatitis. Correlation of (A) Cu with ALT, (B) Cu with BCLC stage, (C) Zn with Alb, (D) Zn with ALP, (E) Zn with Child-Pugh score, (F) Zn with CONUT score, (G) Zn with Cu, (H) the Cu/Zn ratio with BCLC stage, (I) the Cu/Zn ratio with Alb, (J) the Cu/Zn ratio with AST, (K) the Cu/Zn with ALT, (L) the Cu/Zn ratio with ALP, (M) the Cu/Zn ratio with Child-Pugh score, (N) the Cu/Zn ratio with CONUT score, (O) the Cu/Zn ratio with metallothionein, (P) Cu with metallothionein and (Q) Zn with metallothionein. Cu, copper; Zn, zinc; HCC, hepatocellular carcinoma; ALT, alanine aminotransferase; BCLC, Barcelona clinic liver cancer; Alb, albumin; AST, aspartate transaminase; ALP, alkaline phosphatase; CONUT, controlling nutrition status.
(TIFF)

S1 Table Univariate and multivariate analyses in patients with HCC for overall survival by Zn. (DOCX)

S2 Table Univariate and multivariate analyses in patients with HCC for overall survival by Cu. (DOCX)

## Abbreviations

AFP: alpha fetoprotein
ALP: alkaline phosphatase
ALT: alanine aminotransferase
AST: aspartate transaminase
AUC: area under the curve
BCLC: Barcelona clinic liver cancer
BUN: blood urea nitrogen
CONUT: controlling nutrition status
Cu: copper
ECOG: Eastern Cooperative Oncology Group
HBV: hepatitis B virus
HCC: hepatocellular carcinoma
HCV: hepatitis C virus
MT: metallothionein
OS: overall survival
ROC: receiver operator characteristic
Zn: zinc
