# Supplementary figures and images for: Serum copper, zinc and metallothionein serve as potential biomarkers for hepatocellular carcinoma
Source: PLoS One. 2020 Aug 28;15(8):e0237370. doi: 10.1371/journal.pone.0237370 (PMC7455040; doi:10.1371/journal.pone.0237370)

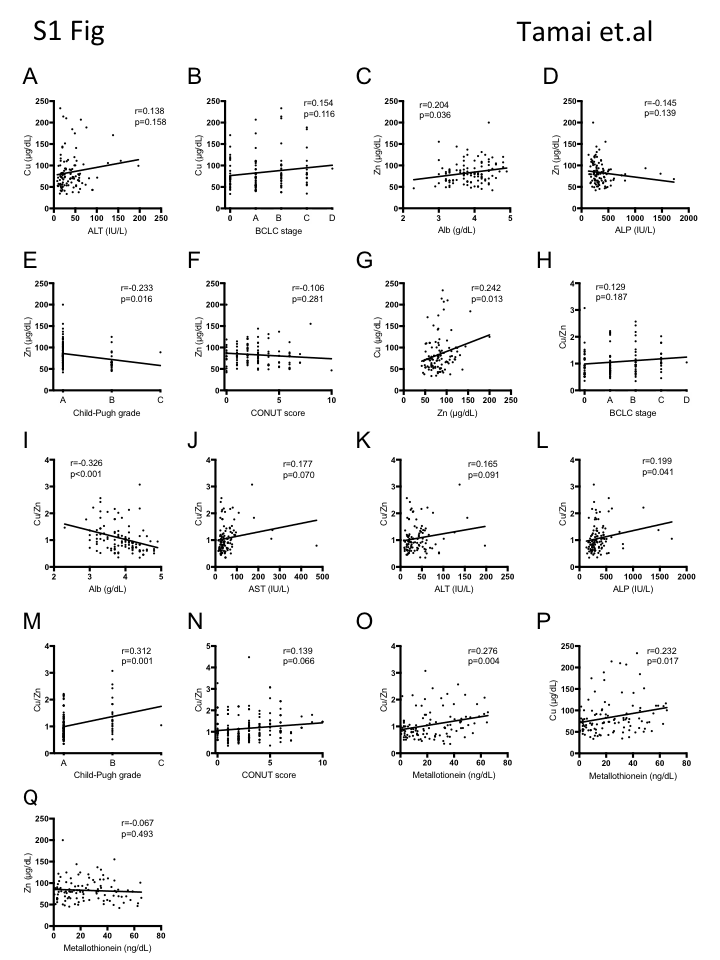

Supplement: S1 Fig — Correlation of (A) Cu with ALT, (B) Cu with BCLC stage, (C) Zn with Alb, (D) Zn with ALP, (E) Zn with Child-Pugh score, (F) Zn with CONUT score, (G) Zn with Cu, (H) the Cu/Zn ratio with BCLC stage, (I) the Cu/Zn ratio with Alb, (J) the Cu/Zn ratio with AST, (K) the Cu/Zn with ALT, (L) the Cu/Zn ratio with ALP, (M) the Cu/Zn ratio with Child-Pugh score, (N) the Cu/Zn ratio with CONUT score, (O) the Cu/Zn ratio with metallothionein, (P) Cu with metallothionein and (Q) Zn with metallothionein. Cu, copper; Zn, zinc; HCC, hepatocellular carcinoma; ALT, alanine aminotransferase; BCLC, Barcelona clinic liver cancer; Alb, albumin; AST, aspartate transaminase; ALP, alkaline phosphatase; CONUT, controlling nutrition status. (TIFF) [file pone.0237370.s001.tiff]

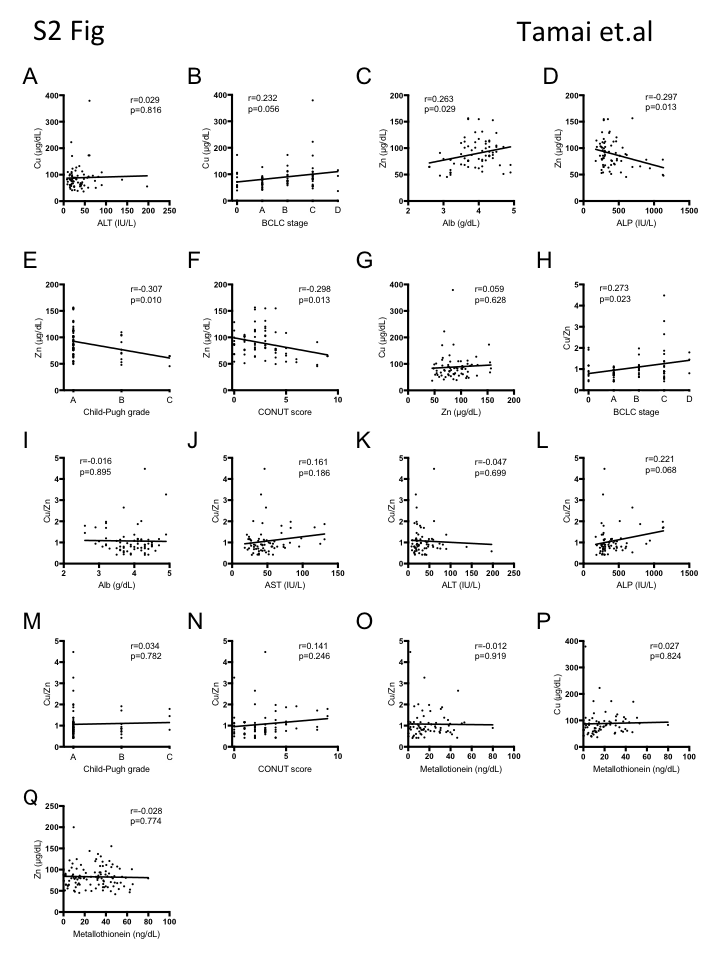

Supplement: S2 Fig — Correlation of (A) Cu with ALT, (B) Cu with BCLC stage, (C) Zn with Alb, (D) Zn with ALP, (E) Zn with Child-Pugh score, (F) Zn with CONUT score, (G) Zn with Cu, (H) the Cu/Zn ratio with BCLC stage, (I) the Cu/Zn ratio with Alb, (J) the Cu/Zn ratio with AST, (K) the Cu/Zn with ALT, (L) the Cu/Zn ratio with ALP, (M) the Cu/Zn ratio with Child-Pugh score, (N) the Cu/Zn ratio with CONUT score, (O) the Cu/Zn ratio with metallothionein, (P) Cu with metallothionein and (Q) Zn with metallothionein. Cu, copper; Zn, zinc; HCC, hepatocellular carcinoma; ALT, alanine aminotransferase; BCLC, Barcelona clinic liver cancer; Alb, albumin; AST, aspartate transaminase; ALP, alkaline phosphatase; CONUT, controlling nutrition status. (TIFF) [file pone.0237370.s002.tiff]
