# Supplementary material for: Serum copper, zinc and metallothionein serve as potential biomarkers for hepatocellular carcinoma
Source: PLoS One. 2020 Aug 28;15(8):e0237370. doi: 10.1371/journal.pone.0237370 (PMC7455040; doi:10.1371/journal.pone.0237370)
Supplement: S1 Table — (DOCX) [file pone.0237370.s003.docx]

**S1 Table. Univariate and multivariate analyses in patients with HCC for overall survival by Zn.**

ECOG, Eastern Cooperative Oncology Group; BCLC, Barcelona clinic liver cancer; AFP, alpha fetoprotein; CONUT, controlling nutrition status; Zn, zinc

|  | Univariate analysis | | | Multivariate analysis | | |
| --- | --- | --- | --- | --- | --- | --- |
|  | HR | 95% CI | P value | HR | 95% CI | P value |
| Age | 1.016 | 0.989-1.044 | 0.248 |  |  |  |
| Gender | 0.968 | 0.538-1.740 | 0.913 |  |  |  |
| ECOG-performance status | 1.709 | 1.005-2.903 | 0.048 | 0.821 | 0.619-1.831 | 0.821 |
| Child-Pugh grade | 1.693 | 1.082-2.649 | 0.021 | 0.787 | 0.481-1.288 | 0.342 |
| BCLC stage | 2.167 | 1.735-2.706 | <0.001 | 2.184 | 1.709-2.792 | <0.001 |
| Creatinine | 1.408 | 0.821-2.417 | 0.214 |  |  |  |
| AFP | 1.000 | 1.000-1.000 | 0.001 | 1.000 | 1.000-1.000 | <0.001 |
| CONUT score | 1.150 | 1.032-1.282 | 0.012 | 1.100 | 0.974-1.288 | 0.123 |
| Metallothionein | 1.010 | 0.997-1.023 | 0.132 |  |  |  |
| Zn | 0.988 | 0.978-0.998 | 0.016 | 0.991 | 0.981-1.002 | 0.100 |
